# Machine-learning-based predictions of imprinting quality using ensemble and non-linear regression algorithms

**DOI:** 10.1038/s41598-023-39374-1

**Published:** 2023-07-26

**Authors:** Bita Yarahmadi, Seyed Majid Hashemianzadeh, Seyed Mohammad-Reza Milani Hosseini

**Affiliations:** 1 Real Samples Analysis Laboratory, Department of Chemistry, Iran University of Science and Technology, Tehran, Iran; 2 Molecular Simulation Research Laboratory, Department of Chemistry, Iran University of Science and Technology, Tehran, Iran

**Keywords:** Biochemistry, Chemistry, Materials science

## Abstract

The molecularly imprinted polymers are artificial polymers that, during the synthesis, create specific sites for a definite purpose. These polymers due to their characteristics such as stability, easy of synthesis, reproducibility, reusability, high accuracy, and selectivity have many applications. However, the variety of the functional monomers, templates, solvents, and synthesis conditions like pH, temperature, the rate of stirring, and time, limit the selectivity of imprinting. The Practical optimization of the synthetic conditions has many drawbacks, including chemical compound usage, equipment requirements, and time costs. The use of machine learning (ML) for the prediction of the imprinting factor (IF), which indicates the quality of imprinting is a very interesting idea to overcome these problems. The ML has many advantages, for example a lack of human error, high accuracy, high repeatability, and prediction of a large amount of data in the minimum time. In this research, ML was used to predict the IF using non-linear regression algorithms, including classification and regression tree, support vector regression, and k-nearest neighbors, and ensemble algorithms, like gradient boosting (GB), random forest, and extra trees. The data sets were obtained practically in the laboratory, and inputs, included pH, the type of the template, the type of the monomer, solvent, the distribution coefficient of the MIP (K_MIP_), and the distribution coefficient of the non-imprinted polymer (K_NIP_). The mutual information feature selection method was used to select the important features affecting the IF. The results showed that the GB algorithm had the best performance in predicting the IF, and using this algorithm, the maximum R^2^ value (R^2^ = 0.871), and the minimum mean absolute error (MAE = − 0.982), and mean square error were obtained (MSE = − 2.303).

## Introduction

**Figure 1:**
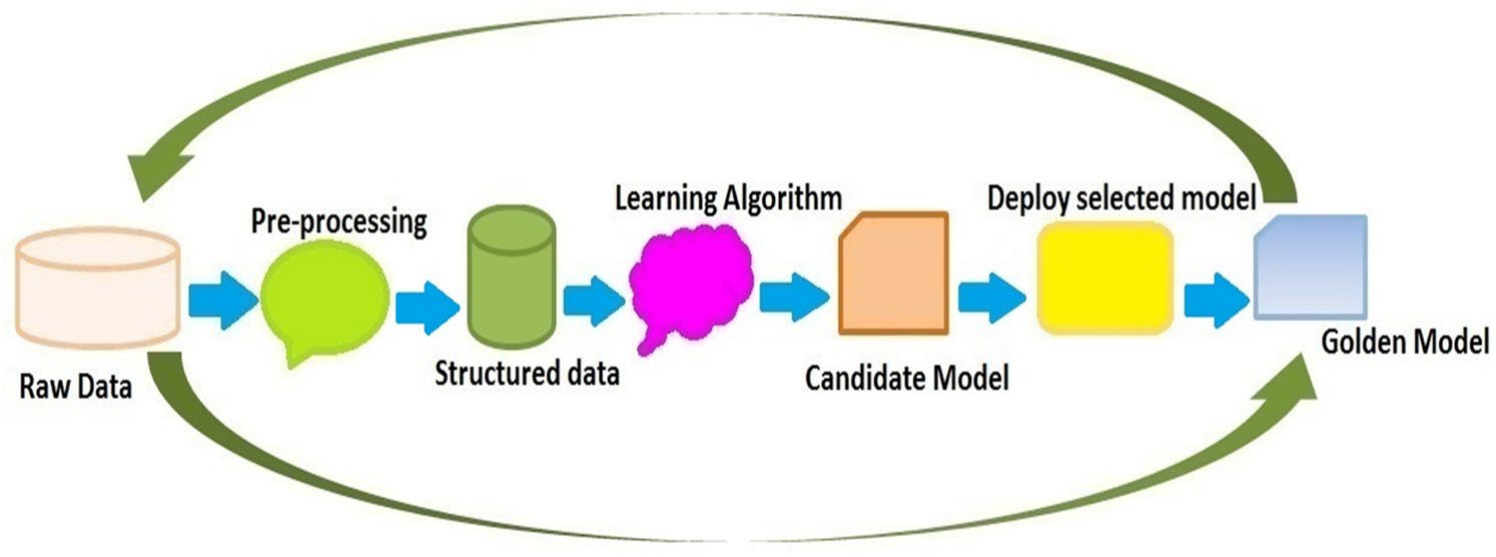
Modeling steps using ML algorithms. These steps include collecting data, preprocessing of the data, applying different algorithms, improving models by cleaning data, or using pipelines.

In the last decade, the desire to use ML has gained popularity due to the large volume data sets that are accessible. The machines are essential for cheap computational processing, usually for accelerated data storage. Therefore, it is possible to quickly, and naturally create models in this field that provide researchers with much larger, more complex, and more detailed information^1^. The Fig. 1 shows the steps of the modeling using ML, like data gathering, pre-processing, using algorithms, improving the model, and creating a final model. Using ML has received attention in various sciences, including chemistry^2^, chemical engineering^3^, physics^4^, geology^5^, pharmacy^6^, medicine^7^, and computer science^8^. Also, it was used in various fields, for example disease diagnosis like cancer^9^, sensor design^10^, drug design^11^, drug delivery^12^, geography^13^, weather prediction^14^, polymers^15^, road control^16^, and traffic detection^17^ has found applications.

The MIPs are synthesis receptor, consisting of polymeric network with selective nano cavities that made based on the shape, size, and functional groups of the template molecule^18^. These polymers have many advantages, such as low cost, stability, easy preparation, and reproducibility^19^. However, the variety of the functional monomers, template, solvents, and synthesis condition like pH, temperature, rate and time of stirring has limited selectivity of imprinting^20^. To overcome these problems, ML can be used to predict the factors that represent imprinting quality. Using ML to predict the IF has many advantages, like short time, low cost, no use of chemicals, no use of equipment, elimination needing to control synthesis conditions such as pH, temperature, stirring rate, and time. Indeed using ML lead to elimination disadvantages of the practical imprinting, and increase precision of imprinting without human error^21^.

The supervised learning is a popular method that widely used in ML. A supervised learning algorithm finds the relationship between training data, and their specific output. This algorithm uses the learned relationship to predict the new inputs^22^. The supervised learning has many advantage, like allowing people to collect data or generate data output from previous experience, helping AI developers optimize performance metrics using expertise, and helping people solve many different types of real-world computational problems^23^.

The regression is a supervised ML algorithm, used to predict the continuous values of the output based on the input. There are three main types of regression algorithms, such as simple linear regression, multiple linear regression, and polynomial regression^24^. This method is mainly used to predict, and find the relationships between features. The regression techniques differ based on the number of the independent features, and the relationship between the independent, and dependent features^25^. The different regression algorithms were used to model imprinted polymer problems. For example, Sunil K.Jha et al. (2014) used the SVR algorithm to predict the response of an advanced MIP-based odor filter, and sensing system for the detection of volatile organic compounds (VOC). According to their results, the accuracy of the model was perfect^26^. Also, Zhenhe Wang (2020) and colleagues designed an elemental quartz crystal microbalance gas sensor based on imprinted polymer, for the detection of volatile organic compounds. They used algorithms like LDA, KNN, PNN, and SVR to predict the response of the sensor. According to their results, using the SVR algorithm leads to create a model with high accuracy^27^. Emma Van de Reydt et al. (2022) successfully used a ridge regression algorithm to create a model to predict the diffusion rate coefficients of various monomers in radical polymerization. The variables studied included boiling point, molecular weight, and dipole moment. Their model is used for monomer like styrene and acrylonitrile, acrylates and methacrylates in general, also accurately predicts the Arrhenius activation parameter and absolute velocity coefficient^28^. Recently, Ahmed Elsonbaty and colleagues successfully optimized the fabrication conditions of four sensors consisting of PVC membranes based on the MIP, using the innovative self-validated ensemble modeling (SVEM). These sensors were prepared using four different experimental designs including SVEM-LASSO, ccentral composite, SVEM-PFWD and SVEM-FWD. Their proposed sensor had a suitable Nernst response^29^.

The ensemble algorithms are one of the powerful learning algorithms for classification, and regression problems. The purpose of these algorithms is to combine multiple weak outputs to achieve a final strong output^30^. Using these algorithms significantly reduces modeling errors, and improves accuracy of the model. Unfortunately, despite improved accuracy, these algorithms are not widely used in polymer development, due to their computational complexity^31^. Chenxi Liu et al. (2022) developed a highly sensitive fluorescence sensor based on molecularly imprinted dual-emitting polymers (dual-em-MIPs) for the detection of pretilachlor in fish, and water samples. They used a RF algorithm to predict sensor responses, and analyze fluorescence images of the samples. Their model perfectly predicted the sensor response^32^.

In this paper, we developed a minimal-error model to predict the IF for various MIPs. Input data including pH, type of the template, type of the functional monomer type, solvent, K_MIP_, and K_NIP_ were obtained in the laboratory. IF values for 100 different MIPs were also calculated. According to the type, distribution, and range of the data, linear algorithms showed a huge prediction error, so they were not investigated. Non-linear regression algorithms like KNN, CART, SVR, and ensemble algorithms such as GB, RF, and ET were used to create a comprehensive model.

## Experimental and methods

### The reagents and materials

All chemical compounds were analytical grade, and all solutions were prepared with double distilled water (DDW). Tetraethyl orthosilicate (TEOS, 98.00%) was purchased from Merck (Darmstadt, Germany). Acrylamide (AA, ≥ 99.00%), Ethanol, Ethylene glycol dimethacrylate (EGDMA, 99.00%), hexadecyltrimethylammonium bromide (CTAB, ≥ 99.00%), hydrochloric acid, and 2, 2′-Azobis(2-methyl propionitrile) (AIBN, 99.00%) were obtained from Sigma-Aldrich (www.sigmaaldrich.com, St. Louis, MO, USA). All templates of pure powder were purchased from RazakPharma Company (www.RazakPharma.com, Tehran, Iran).

### The apparatus

A scanning electron microscope (Model: GeminiSEM 460) was used to observe the morphology of the surface of MIP before and after washing with a suitable solvent. The absorbance spectra were obtained using a UV-1600 spectrometer.

### The synthesis of MIP

The polymers in a typical process were synthesized as will be explained below: First, 1 ml of TEOS, 0.7 ml DDW, 1 ml ethanol, and 1 mL of hydrochloridric acid (0.2 mol L^-1^) were mixed and stirred for 22 min. Then, 0.1 mg of AA (functional monomer), 2mg of AIBN, and 100 mg of EGDMA were added, and the mixture stirring for three hours at room temperature. Subsequently, the template molecule, was dissolved in 25 ml of hydrochloric acid (2 mol L^-1^), in a beaker to reach pH = 6.2. Then the solution was stirred for 15 min at 42 °C to prepare the preassembly solution. At the end of the procedure, 5 ml ethanol, and 0.32 g CTAB were added to the mixture, and the mixture was kept stirring for one hour at room temperature. Also, for comparison, non-imprinted polymer (NIP) was prepared using the same procedure only without the addition of a template molecule in the polymerization process.

The MIP for one hundred templates was synthesized, like Naproxen, Nicotinamide, Ibuprofen, Cholesterol, Bisphenol A, (S)-Nilvadipine, d-Chlorpheniramine, Cinchonine, Nicotine, Salicylic aldehyde, folic acid, etc. Also, different functional monomers such as acrylic acid, acrylamide, methyl methacrylate, allylthiourea, 4-VBA, L-Val, 4-VP, 2-HEMA, APTES, and 2-MAOEP were used. During synthesis, pH was optimized using buffers (H_3_PO_4_/NaHPO_4_^–2^), and optimal pH was considered an essential factor in synthesis of the MIP^33,34^.

### Dataset

The main components of the MIPs are functional monomer, template, cross-linker, and solvent. If during synthesis pH changes, the structure of compounds that have proton or hydroxyl groups will change, and this structural change affects the imprinting quality, then pH is an essential factor in the synthesis MIPs. The solvent dissolves other reagents, so the type and volume of the solvent are significant factors. The distribution coefficient of NIP, and MIP is related to the imprinting quality. The high selectivity coefficient indicates strong imprinting, and the low selectivity coefficient indicates poor imprinting.

The difference between the concentration of the template molecules in solution after, and before absorption by MIP, leads to the determination of distribution coefficient (K) as per Eq. (1) that in this equation, m is the mass of the polymer, V is the volume of the solution., C_i_ is the initial concentration of the template molecules, and C_f_ is the equilibrium concentration of the template molecules. The monomer-template complex stability criteria (IF) was calculated using the following Eq. (2):

1 $${\text{K}} = \left( {{\text{C}}_{{\text{i}}} - {\text{C}}_{{\text{f}}} } \right){\text{V}}/{\text{mC}}_{{\text{f}}}$$

2 $${\text{IF}} = {\text{K}}_{{{\text{MIP}}}} /{\text{K}}_{{{\text{NIP}}}}$$

The dataset plays a vital role in modeling. In this study dataset was obtained experimentally in the laboratory. Different features, including, the type of the template, K_MIP_, K_NIP_, pH, the type of the functional monomer, the type, and volume of solvent were used as input, and IF as output. The descriptor of the template, functional monomer, and solvent, respectively, was, topological polar surface area, average dipole moment, and XLogp3 that were obtained from the Pubchem site. The number of the samples was one hundred, and to get a comprehensive model, we used different template molecules, and various algorithms. Python 3.11.1 software was used for modeling.

### The modeling using non-linear regression, and ensemble algorithms

The performance of the model is influenced by three steps, including feature selection, algorithm selection, and cross-validation. These steps are critical, and if these three steps are not performed carefully, it will lead to creating a model with an error. Therefore, these steps must be done carefully. The features will have a significant effect on the performance, accuracy, and efficiency of the model. Perhaps the most crucial part of data mining operations and modeling is the feature selection method, because unrelated or somewhat related features reduce system performance. Implementing feature selection methods is the first, and important step in designing intelligent learning systems. Also, when the dimension of the data feature space is vast, and we are faced with the dimension problem, using the appropriate feature selection method reduces the “computational costs” required for optimal system training. Feature selection leads to dimensionality reduction by removing irrelevant and repetitive features. Due to we are facing a regression problem, the mutual information feature selection method was used. The mutual information method uses the application of information gained from the dataset (typically used in the construction of decision trees) to feature selection. In this method, mutual information is calculated between two features, and it measures the reduction in uncertainty for one feature given a known value of the other feature.

Regression is a predictive modeling technique that examines the relationship between a dependent and an independent variable. To accurate modeling, different algorithms, like SVR, CART, KNN, GB, ET, and RF were used. The SVR algorithm is a method where you plot the raw data as points in an n-dimensional space where n is the number of features you have. Each feature is tied to a specific coordinate on the page, making data classification easy. The KNN algorithm is a non-parametric statistical method used for statistical classification and regression. In the regression case, the average of the values obtained from K is its output. The CART algorithm is an algorithm that is needed to build a decision tree based on the Gini impurity index. In this algorithm, to progress from the observations on the samples represented by the branches, and to the desired target value represented by the leaves, conclusions are drawn. The GB algorithm consists of three elements, including a loss function for optimization, a poor learner for predictions, and a collective model for adding a weak learner to minimize the loss function. The GB algorithm is greedy and can quickly over fit the training data set. Therefore, regularization methods can penalize different parts of the amplification algorithm. The RF Algorithm, includes several decision trees in different subsets of the data set and takes an average to improve the prediction accuracy of that data set. Instead of relying on a decision tree, RF predicts the prediction from each tree based on the majority of votes and considers the final result as the output. The ET algorithm is an ensemble supervised method that uses decision trees. This method is similar to the RF algorithm but can be faster. This algorithm, like the RF algorithm, creates many decision trees, but the sampling for each tree is random, without replacement.To achieve a model that predicts the IF value with minimum error, all these algorithms were applied to the data.

Another essential parts of any ML modeling, is model validation. The process of the data separation involves dividing a data set into two or three subsets. Evaluating the model provides essential information about the performance. To divide the dataset into the test and train data, the shuffle split cross validation was used (n-splits = 10.000, test-size = 0.300, random state = 1.000). Also, to increase the accuracy of the model and prevent leakage of the test data to the training data, Pipelines were used. After determining the performance of the algorithms, the hyper parameters of the best algorithm (GB algorithm) were tuned.

### Ethics approval and consent to participate

Authors consciously assure that for the manuscript, the following is fulfilled:

1. This material is the author’s original work, which has not been previously published elsewhere.
2. The paper is not currently being considered for publication elsewhere.
3. The paper reflects the author's research and analysis in a truthfully and completely manner.
4. All authors have been personally and actively involved in substantial work leading to the paper, and will take public responsibility for its content.

I agree with the above statements and declare that this submission follows the guidelines for Authors and the Ethical Statement.

## The results and discussions

### The surface evaluation

**Figure 2:**
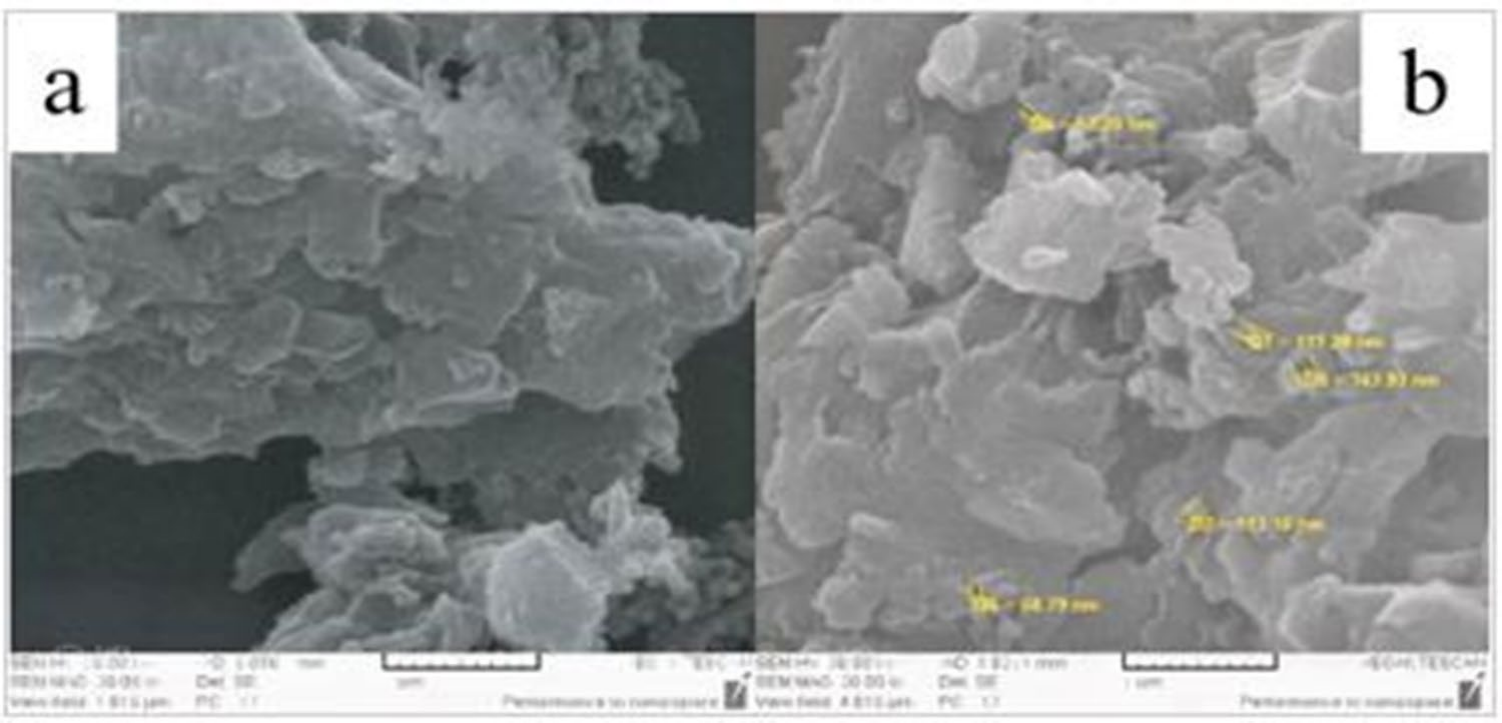
The SEM image of the surface of MIP before (**a**) and after (**b**) washing with suitable solvent. After washing the MIP with a suitable solvent capable of removing the template molecules from the network, holes the size of the template molecule remained.

The Fig. 2 shows an SEM image of the surface of the MIP before (a) and after (b) washing with suitable solvent. It is clear that after washing MIP, template molecules leave the polymeric network, and cavities remain base on the functional group, shape, and size of the template molecule. The optimal synthesis conditions of the MIP, leads to excellent stability, and the MIP can be reused for extraction template molecules.

### The data recognition

Data recognition is the first, and most important step in ML. The descriptive statistics can give us a great insight into each feature, and by using it, we can review more summaries of the features in minimum time. The use of descriptive statistics, eight characteristics, like number, average, maximum, minimum, standard deviation, %25, %50, and %75 for each feature was obtained. The results of the statistical analysis of the data are shown in Table 1.

**Table 1:** The results of the statistical data analysis, including number, average, maximum, minimum, standard deviation, %25, %50, and %75 for each feature.

Statistical parameter	Template	Monomer	K_MIP_	K_NIP_	pH	Solvent	Volume	IF
Count	100.000	100.000	100.000	100.000	100.000	100.000	100.000	100.000
Mean	66.378	2.121	8.028	2.577	6.461	0.221	88.822	4.0193
Std	37.999	0.757	12.193	4.388	1.107	1.102	18.439	4.062
Min	0.000	1.350	0.000	0.000	2.000	2.100	50.000	0.800
25%	38.400	1.780	0.900	0.362	6.800	0.000	83.750	1.427
50%	56.750	1.780	3.325	1.255	7.000	0.000	100.000	2.410
75%	97.600	2.240	10.550	2.737	7.000	0.000	100.000	4.790
Max	156.000	4.530	70.600	34.000	7.000	3.900	100.000	23.000

### The feature selection

**Figure 3:**
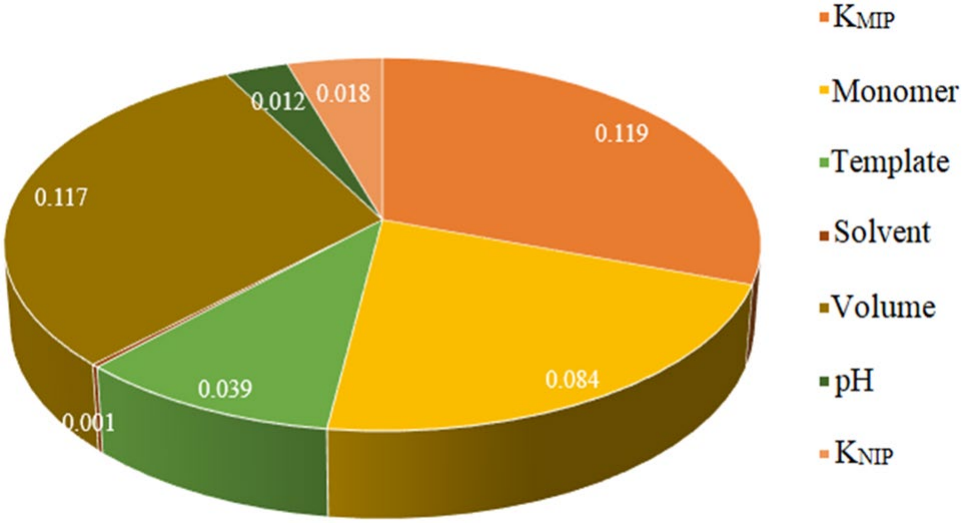
The result of using the mutual information feature selection method for determination important feature affected IF. The K_MIP_, volume of the solvent, the type of the functional monomer, and the type of the templates had the significant impact on the IF. Features like K_NIP_, pH, and the type of solvent had the less effect on the IF.

The mutual information feature selection method was used to determine the important features affecting the IF. The result of the using this method is that assigns a score to each feature. A higher score shows that feature have more effect on the output. The results of using mutual information feature selection method are shown in Fig. 3. The K_MIP_, the volume of the solvent, the type of the functional monomer, and the type of the templates had the significant impact on the IF. Features such as K_NIP_, pH, and the type of solvent had the less effect on the IF.

### The non-linear regression, and ensemble algorithms

After standardizing the data, and applying feature selection, and cross-validation, algorithms, including KNN, SVR, CART, GB, ET, and RF were used. The pipeline was used to provide leaking training data to test data, and vice versa. The results of using different algorithms are shown in Table 2. The use of ensemble algorithms lead to improve the performance of the model .Among the regression algorithms, the CART algorithm had the highest value of R^2^ (R^2^ = 0.645), the lowest error (MAE = − 1.966, MSE = − 13.811). The minimum accuracy of the model using the regression algorithms was related to SVR (R^2^ = 0.045, MSE = − 38.735, MAE = − 3.183). The GB algorithm performed better than the other algorithms in predicting IF, and it had the highest R^2^ (R^2^ = 0.859893), lowest error (MAE = − 0.980446, MSE = − 7.133007). Therefore, the GB algorithm was selected as the best algorithm for predicting IF, and adjusted in the next step.

**Table 2:** The results of using the non-linear regression, and ensemble algorithms.

Number	Algorithms	R^2^	MAE	MSE
1	KNN	0.204 (0.269)	− 2.948 (0.304)	− 28.850 (8.762)
2	CART	0.645 (0.079470)	− 1.966 (0.234)	− 13.811 (6.938)
3	SVR	0.045 (0.065)	− 3.183 (0.477)	− 38.735 (17.120)
4	GB	0.862 (0.077)	− 0.980 (0.512)	− 7.133 (6.503)
5	ET	0.727 (0.161)	− 1.666 (0.244)	− 9.671 (5.328)
6	RF	0.743 (0.174)	− 1.865 (0.309)	− 10.331 (5.934)

### The gradient boosting algorithm tuning

The n_estimators is a suitable candidate parameter for adjustment, and increasing the GB algorithm performance. The default number of boosting steps to perform is 100. A larger number of reinforcement steps results in better performance of the algorithm, but does not increase the training time. To tune the n_estimators, their values were defined from 50 to 500 in steps of 50. The results are shown in Table 3. By tuning the n_estimators on 250, the best performance of the model was obtained, and the model had the maximum value of R^2^ (R^2^ = 0.870021), and the minimum error (MAE = − 0.982562, MSE = − 2.303335).

**Table 3:** The results of the tuning the GB algorithm, and the n_estimators hyper parameter values.

n_estimators	R^2^	MAE	MSE
50	0.645 (0.216)	− 0.996 (0.417)	− 2.510 (3.271)
100	0.709 (0.138)	− 0.915 (0.417)	− 2.352 (3.070)
150	0.704 (0.139)	− 0.902 (0.408)	− 2.326 (2.923)
250	0.871 (0.737)	− 0.982 (0.407)	− 2.303 (2.965)
300	0.704 (0.139)	− 0.902 (0.408)	− 2.326 (2.923)
350	0.703 (0.139)	− 0.902 (0.408)	− 2.326 (2.921)
400	0.703 (0.139)	− 0.902 (0.408)	− 2.326 (2.920)
45O	0.703 (0.139)	− 0.902 (0.407)	− 2.326 (2.919)
500	0.703 (0.139)	− 0.902 (0.408)	− 2.326 (2.919)

### The residual and prediction error plots

**Figure 4:**
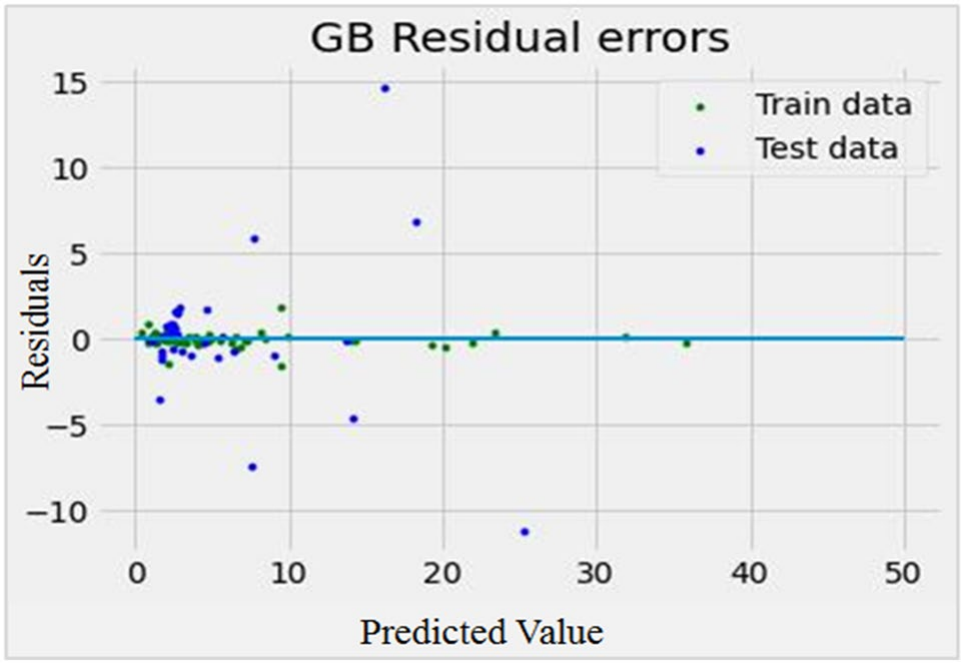
The residual plot of the model in optimal conditions, using the GB algorithm after tuning the n-estimators (n-estimators = 250). Because the training, and test data are closer, the output is more accurately predicted.

**Figure 5:**
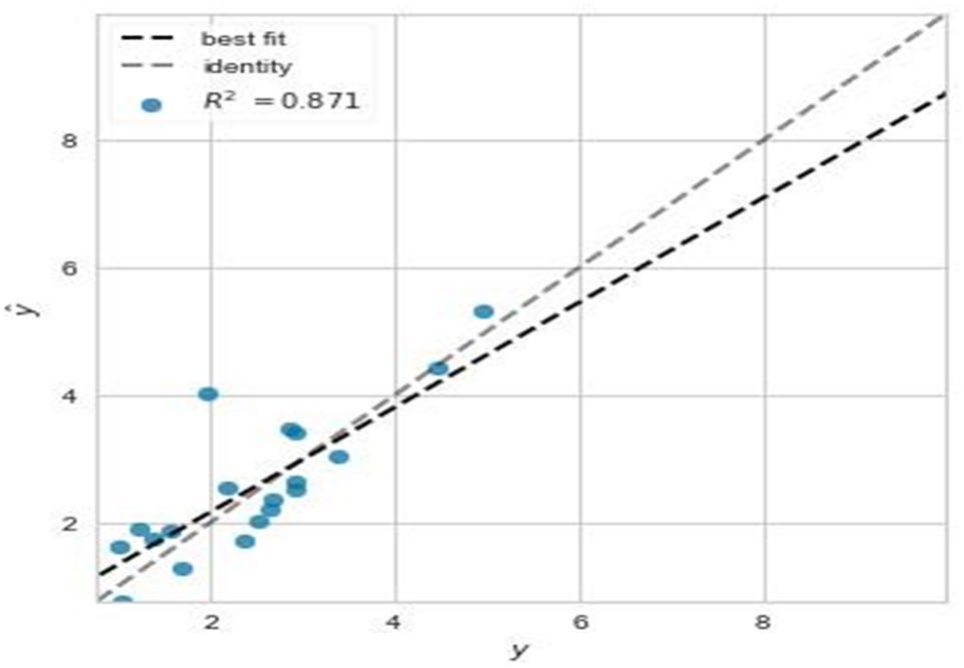
The prediction error plot of the model using the GB algorithm after tuning the n-estimators (n-estimators = 250). The closer the best fit, and the identity line are, the more accurate the prediction. Assuming no errors, the two lines almost coincide.

After setting the n_estimator value, plots of residuals, and prediction errors were used to assess the error of the model. The residual is a measurable error that depends on the estimates obtained from the population parameter. A residual plot shows the relationship between a particular independent feature, and its output, given the presence of other independent features in the model. This plot of the model is shown in Fig. 4. A regression prediction error plot is a qualitative measure of how a model predicts a response. In determining the prediction error plot, the x_train variable as an independent feature, and the y_train feature as a dependent feature plays a role in the model. This plot of the model is shown in Fig. 5. After tuning the GB algorithm, and the n_estimator values, the maximum R^2^ value of the model was obtained (R^2^ = 0.861), and the model has a remarkable ability to predict the IF. Both Figs. 4, and 5 were drawn by matplotlib library of python software.

### Application of the model

The last criterion to evaluate the performance of the model, is its applicability in predicting outputs which their real value is known. Therefore, the use of the model in IF prediction was done for thirty different samples, and the results are shown in Table 4 .In Table 4, the actual and predicted IF values are close with a slight difference. This shows the excellent performance of the model in predicting IF with minimum error.

**Table 4:** The application of the model in predicting the IF for thirty different samples.

IF real	IF predicted	IF real	IF predicted	IF real	IF predicted
1.800	1.680	9.980	9.870	1.920	1.860
2.170	2.140	4.950	4.860	1.080	1.010
7.100	6.790	1.380	1.290	1.220	1.020
2.910	1.950	1.140	1.080	2.530	2.480
2.910	2.760	1.690	1.390	2.770	2.650
1.690	1.440	2.470	2.090	1.80	1.750
1.990	1.790	1.360	1.020	2.150	2.080
1.000	1.010	1.050	1.010	7.300	7.010
1.120	1.090	1.460	1.400	1.140	1.050
5.890	5.760	2.960	2.890	4.720	4.690

## Conclusion

Using ML to predict IF, and determine optimal synthetic conditions lead to reduce costs, improve accuracy, increase reproducibility, decrease human error, increase speed, save time, eliminate consume chemical reagents, and no needing devices, and equipment. To create an accurate model, different algorithms such as CART, KNN, SVR, RF, ET, and GB, and the pipelines were used. The ensemble algorithms showed more accuracy in predicting the IF than non-linear regression algorithms, because these algorithms combine several weak outputs to achieve an accurate output. The minimum accuracy of the model using non-linear regression algorithms was related to SVR algorithm (R^2^ = 0.045, MAE = − 3.183, MSE = − 38.735). The CART algorithm had the maximum prediction accuracy among the non-linear regression algorithms (R^2^ = 0.645, MAE = − 1.966, MSE = − 13.811). The results showed that the GB algorithm, after tuning the n_stimators hyper parameter, had the maximum accuracy of the model (R^2^ = 0.871, MAE = − 0.982, MSE = − 2.303). The change in the accuracy of the model when using different algorithms, depends on the algorithm type, and data distribution. Another important factor that affects model accuracy, is the correlation between features, and output. Furthermore, the type of the feature selection method used for modeling is effective in increasing or decreasing the final accuracy of the model. The most important challenge that researchers face for modeling, is the preparation of literature or experimental data sets. So, the only limitation in this work was the preparation of the experimental data set. It is worth noting that after producing these data in the laboratory once and performing modeling with precision, there is no need for practical work, waste of time and money, and the produced models can be used for different samples and under different syntheses. In fact, using this model, the IF of different template molecules can be predicted with high accuracy and minimum error. Although the use of GB algorithms in chemistry prediction problems is still new, the results presented in this study are very encouraging.

## Data Availability

The data and materials that support the findings of this study are available from the corresponding author, upon reasonable request.
